# Complex IgE sensitization patterns in ragweed allergic patients: Implications for diagnosis and specific immunotherapy

**DOI:** 10.1002/clt2.12179

**Published:** 2022-07-05

**Authors:** Maria‐Roxana Buzan, Lauriana‐Eunice Zbîrcea, Pia Gattinger, Elijahu Babaev, Frank Stolz, Rudolf Valenta, Virgil Păunescu, Carmen Panaitescu, Kuan‐Wei Chen

**Affiliations:** ^1^ Center of Immuno‐Physiology and Biotechnologies, Department of Functional Sciences Victor Babes University of Medicine and Pharmacy Timisoara Romania; ^2^ OncoGen Center Pius Brinzeu County Clinical Emergency Hospital Timisoara Romania; ^3^ Department of Pathophysiology and Allergy Research, Division of Immunopathology, Center of Pathophysiology, Infectiology and Immunology Medical University of Vienna Vienna Austria; ^4^ Biomay AG Vienna Competence Center Vienna Austria; ^5^ Laboratory for Immunopathology, Department of Clinical Immunology and Allergology Sechenov First Moscow State Medical University Moscow Russia; ^6^ Karl Landsteiner University of Health Sciences Krems Austria; ^7^ NRC Institute of Immunology FMBA of Russia Moscow Russia

**Keywords:** allergy diagnosis, cross‐reactive carbohydrate determinants, IgE reactivity, ragweed allergy, sensitization profile

## Abstract

**Background:**

Ragweed (*Ambrosia artemisiifolia*) is one of the most important allergen sources, worldwide, causing severe respiratory allergic reactions in late summer and fall, in sensitized patients. Amb a 1 has been considered as the most important allergen in ragweed but 12 ragweed pollen allergens are known. The aim of our study was to investigate IgE reactivity profiles of ragweed allergic patients and to associate them with clinical symptoms.

**Methods:**

IgE sensitization profiles from clinically well‐characterized ragweed allergic patients (*n* = 150) were analyzed using immunoblotted ragweed pollen extract. Immunoblot inhibition experiments were performed with two Amb a 1 isoforms and CCD markers and basophil activation experiments were performed with IgE serum before and after depletion of Amb a 1‐specific IgE.

**Results:**

By IgE‐immunoblotting 19 different IgE reactivity patterns with and without Amb a 1‐sensitization were found. The majority of patients (>95%) suffered from rhino‐conjunctivitis, around 60% reported asthma‐like symptoms and about 25% had skin reactions. Patients with complex IgE sensitization profiles tended to have more clinical symptoms. Serum with and without Amb a 1‐specific IgE induced basophil activation.

**Conclusions:**

Ragweed pollen allergic patients exhibit complex IgE reactivity profiles to ragweed allergens including Amb a 1 isoforms and cross‐reactive carbohydrates indicating the importance of Amb a 1 isoforms and additional allergens for diagnosis and allergen‐specific immunotherapy of ragweed allergy.

## 1 INTRODUCTION

*Ambrosia artemisiifolia*, also known as common or short ragweed is an invasive plant originating from North America and now occurring worldwide, including certain parts of Europe and Asia.^1^, ^2^, ^3^

In Europe, ragweed became domestic in the Pannonian Plain and parts of the surrounding countries such as Austria,^4^ Slovakia,^5^ Serbia,^6^, ^7^ Croatia,^6^, ^8^ Slovenia,^6^ Hungary^9^ and Romania,^6^, ^10^, ^11^ in the Rhône Valley and northern Italy.^12^, ^13^ In these areas high sensitization rates among atopic patients could be observed, 60% in Hungary,^14^ 47% in the Rhône Valley area,^15^ and 70% in northern Italy.^16^

Ragweed pollen allergy represents a major health issue in ragweed infested areas due to the high sensitization rates and induction of severe respiratory symptoms. Asthma symptoms were reported in 23.7% of ragweed allergic patients^14^ and ragweed pollen was found to induce asthma twice as often as other types of pollen.^17^ So far, ragweed occurrence is limited to some areas in Europe but due to climate change and urbanization, it may continue spreading.^13^, ^18^, ^19^

Amb a 1, formerly antigen E, is a major allergen^20^ with distinct isoforms, which varies in amino acid sequences and IgE reactivity.^21^, ^22^ The most expressed and IgE reactive isoforms are Amb a 1.01 (396 amino acids)^23^ and Amb a 1.03 (397 amino acids)^24^ which share a 76.26% homology.^2^, ^22^ Amb a 1 has been considered as the clinically most important allergen of ragweed.^25^ Allergen‐specific immunotherapy (AIT) based on purified Amb a 1 or Amb a 1‐derivatives has been considered to replace ragweed pollen extract for AIT.^26^, ^27^ This major allergen belongs to the pectate lyase allergen family and shows varying IgE cross‐reactivity with Art v 6 (mugwort), Cup a 1/Jun a 1 (cypress) and Cry j 1 (cedar).^28^

But meanwhile, other 11 ragweed pollen allergens have been described in the WHO/IUIS allergen nomenclature database.^29^ Among them, Amb a 11, a cysteine protease sharing homology with other allergens from the same protease family, such as Act d 1 (kiwi fruit), Ana c 2 (pineapple), and Der f/Der p 1 (dust mites),^2^ was also classified as a major ragweed pollen allergen.^30^ Others are minor allergens such as a small protein (Amb a 5), two plastocyanins (Amb a 3, Amb a 7), a defensin (Amb a 4) related to Art v 1 from mugwort^31^ and a recently discovered enolase (Amb a 12). Additional cross‐reactive allergens in ragweed pollen are profilin (Amb a 8), polcalcins (Amb a 9, Amb a 10) and a lipid transfer protein (Amb a 6).^2^

Accordingly, ragweed pollen is a complex allergen source containing several isoforms of Amb a 1 and other allergens with varying cross‐reactive potential. Currently, commercially available component resolved diagnosis offers only natural or recombinant Amb a 1 and Amb a 4. Whether these allergens alone are sufficient for an accurate diagnosis remains unclear. Compared with birch pollen allergy where 8 allergens were described but only one allergen is dominant (Bet v 1),^32^ one or few allergens are sufficient for an accurate diagnosis. But considering house dust mite (HDM) allergy where three allergens are serodominant (Der p 1, Der p 2 and Der p 23) and three other allergens (Der p 5, Der p 7 and Der p 21) are considered as clinically relevant,^33^ all these allergens have to be included in order to have an accurate diagnosis. In the case of ragweed pollen allergy, there is no clear information regarding the IgE binding profile. Therefore, no conclusion can be made whether Amb a 1 only is sufficient for the diagnosis of ragweed pollen allergy.

We aimed to investigate IgE reactivity profiles of ragweed allergic patients, by performing an extensive IgE‐immunoblot study combined with IgE inhibition experiments with purified ragweed allergens and basophil activation experiments using sera from clinically well‐characterized patients. The importance and novelty of this study lie in the better illustration of the heterogeneity and complexity of ragweed pollen allergy. Our results reveal several different IgE sensitization profiles indicating that molecular forms of diagnostics and immunotherapy may need to include Amb a 1 isoforms and additional ragweed pollen allergens.

## 2 MATERIALS AND METHODS

Only essential information was included in this section, more details can be found in Supporting Information S1.

### 2.1 Patients' sera

Patients included in this study were recruited from an allergy center in Timisoara, Romania, where they were clinically evaluated by an allergist, based on ARIA^34^ and GINA^35^ criteria. Further allergy tests were performed based on the anamnesis to confirm the suspected sensitization. From this patient pool, 150 ragweed‐allergic patients were included in our study with a case history indicative of seasonal ragweed allergy, positive skin prick test or/and serum tests for ragweed‐specific IgE.

All recruited patients reported severe symptoms only during the ragweed pollen season although they were not all monosensitized to ragweed.

Serum samples were collected after written informed consent was obtained. The study was approved by the Local Ethics Commission of Scientific Research of the Pius Brinzeu Emergency County Hospital Timisoara (Ethical approval number 102, 10.01.2017).

### 2.2 Pollen extract and allergens

Aqueous ragweed pollen extract, natural Amb a 1.01 (nAmb a 1.01), recombinant Amb a 1.03 (rAmb a 1.03), recombinant glycoprotein HHM2 (horse heart myoglobin) and recombinant Der p 2 were produced as described.^22^, ^36^, ^37^, ^38^

### 2.3 ImmunoCAP measurement for CCDs

Carbohydrate‐specific IgE levels were quantified by ImmunoCAP measurements using biotinylated ProGlycAn P (HÄMOSAN Life Science Services GmbH, Austria) coupled to Streptavidin ImmunoCAPs (o121) (Thermo Scientific, Phadia AB, Uppsala, Sweden).^37^

### 2.4 Western‐blot, SDS‐PAGE and immunoblot inhibition

Ragweed pollen extracts, separated under reducing conditions by SDS‐PAGE were blotted onto nitrocellulose membranes and incubated with patients' sera, diluted 1:10. Bound IgE was detected with ^125^I‐labeled anti‐human IgE (BSM Diagnostica, Vienna, Austria) and visualized by autoradiography.^39^ Serum from a non‐allergic individual and buffer without serum were used as negative controls.

For the comparison between ragweed pollen extract and the Amb a 1 isoforms, four concentrations of extract (20 μg, 10 μg, 5 μg, 1 μg) and 1 μg of nAmb a 1.01 and rAmb a 1.03 were separated by 18% SDS‐PAGE followed by Coomassie Brilliant Blue staining.

For IgE immunoblot inhibitions, 1:10 diluted sera from ragweed allergic patients and individuals exclusively sensitized to CCDs (PC1‐3, Figure 4) were pre‐incubated with 5 μg/ml of nAmb a 1.01, rAmb a 1.03 and 5 μg/ml of HHM2 and ProGlycAn P, for CCD positive patients. HHM2 is the first recombinant carbohydrate marker that resembles the IgE epitope spectrum of N‐linked glycans of insect venoms, plants and even mites.^37^ Pre‐incubation with rDer p 2, an MD‐2 like lipid‐binding protein^40^ not present in pollens and with no cross‐reactive to any ragweed pollen allergens, was used as a negative control. Pre‐adsorbed sera were incubated with nitrocellulose‐blotted ragweed pollen extract and bound IgE was detected as described above.

### 2.5 Removal of Amb a 1.01 and 1.03‐specific IgE

Sera from three Amb a 1 allergic patients (54, 81, 89), co‐sensitized to other ragweed allergens and negative to CCDs, were repeatedly incubated, first on nAmb a 1.01 coated plates, then on rAmb a 1.03 coated plates (5 μg/ml), following the schedule: 2 × 3 h, overnight, 4 × 2.5 h, overnight, 2 × 3 h, overnight, 3 h, at 4°C. IgE antibody removal was confirmed by ELISA.

### 2.6 Basophil activation assays

**FIGURE 1:**
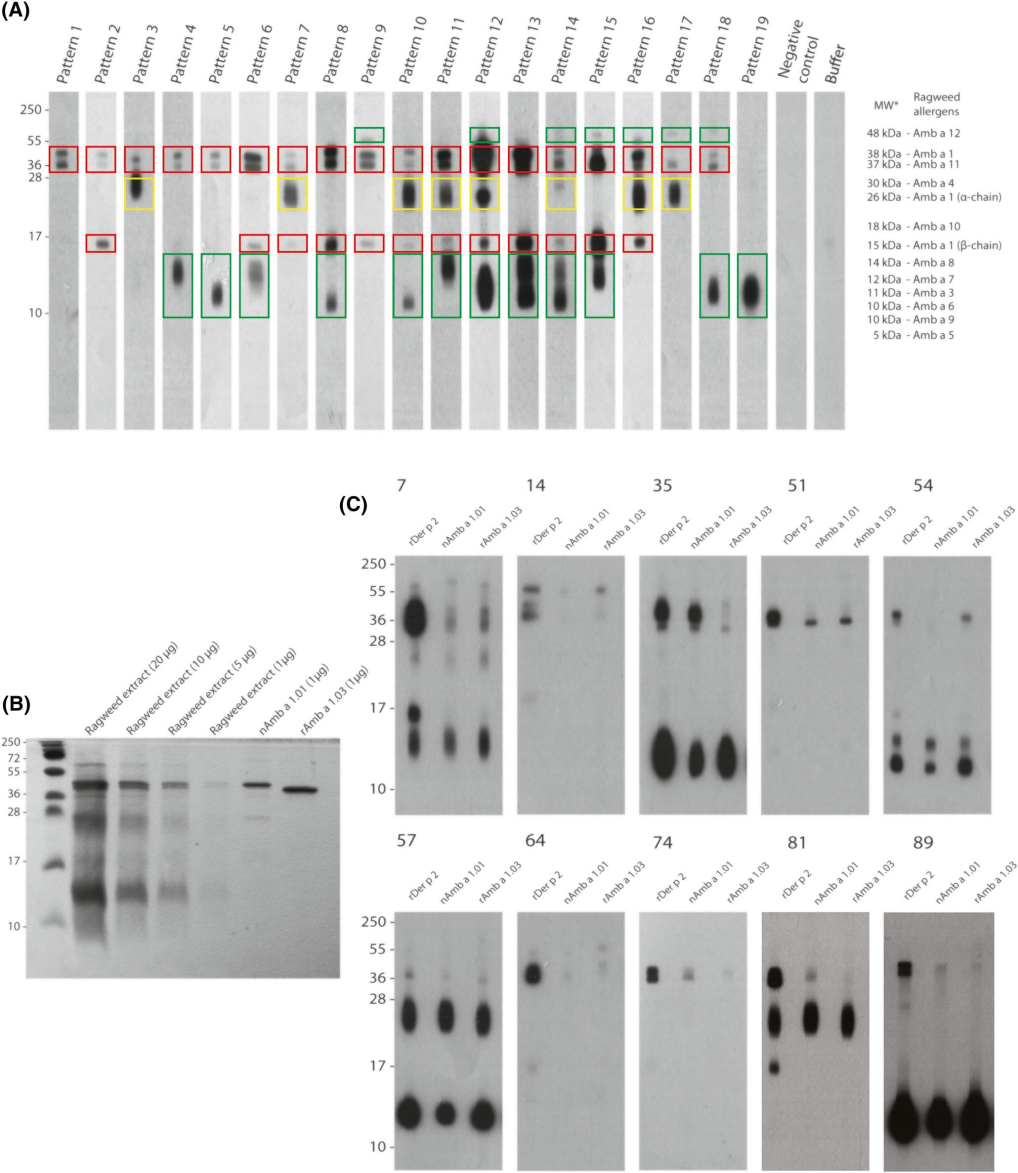
IgE reactivity patterns of representative ragweed allergic patients to nitrocellulose‐blotted ragweed pollen extract. (A) Immunoblot results showing 19 different representative IgE reactivity patterns (P1‐P19) observed in 130 ragweed allergic patients with a negative response to CCDs in ImmunoCAP. Serum from a non‐allergic individual (Negative control) and buffer without serum (Buffer) were used as negative controls. The bands inhibited by at least one of the Amb a 1 isoforms were marked with red frames, the bands only partially inhibited by the two Amb a 1 isoforms were marked with yellow frames, the bands which were not inhibited by Amb a 1 were marked with green frames. Possible ragweed allergens corresponding to the molecular weight (MW) of the bands are displayed on the right. * MW as reported in the literature^2^. (B) Four different concentrations of ragweed extract were loaded on SDS‐PAGE compared to the two Amb a 1 isoforms, natural Amb a 1.01 (nAmb a 1.01) and recombinant Amb a 1.03 (rAmb a 1.03). (C) Immunoblot inhibitions. Sera from 10 ragweed allergic patients (7, 14, 35, 51, 54, 57, 64, 74, 81, 89) with different IgE sensitization patterns were preadsorbed with rDer p 2, nAmb a 1.01 or rAmb a 1.03 and tested for IgE reactivity to nitrocellulose‐blotted ragweed pollen extract. Molecular weight (kDa) markers are indicated on the left

Rat basophil leukemia cells transfected with human FcεRI (RS‐ATL8)^41^ were used for mediator release assays. RBL cells were passively sensitized with sera before and after depletion of Amb a 1.01 and 1.03‐specific IgE, then stimulated with serial dilutions of ragweed pollen extract (0.1 ng/ml–10 μg/ml), nAmb a 1 and rAmb a 1.03 (0.01 ng/ml–1 μg/ml) and β‐hexosaminidase release was measured. A tenfold higher concentration of ragweed extract, compared to Amb a 1 was used according to pilot experiments (SDS‐PAGE, Figure 1B) and earlier reports estimating that Amb a 1.01 and 1.03 represent 11.8% and 6.6%, respectively, of ragweed pollen proteins.^42^ Buffer without allergens was used as a negative control.

### 2.7 Statistical analysis

Statistical analysis was performed using SPSS version 25.0 (IBM Corp.). Differences between patients with only Amb a 1 sensitization and patients with Amb a 1 and/or other ragweed pollen allergens sensitization were evaluated by Chi‐square test and were considered statistically significant if the *p* value was ≤0.05.

## 3 RESULTS

### 3.1 Clinical characterization of ragweed pollen allergic patients

In this study, 150 ragweed allergic patients were analyzed. Symptoms reported during the ragweed pollen season were recorded and no significant differences between mono‐ and polysensitized patients were observed (Table 1). The most frequent combination of symptoms was rhinitis + conjunctivitis + asthma‐like symptoms (39.3%) (Table 1). Clinical symptoms and other sensitizations are shown in Table S1.

**TABLE 1:** Demographic and clinical characterization of the study population

Total number of patients	150
Age (mean, range)	35.9/18–61 years
Sex (male/female)	100/50

	Total		Ragweed monosensitized		Polysensitized		*p* value
	No. of patients	%	No. of patients	%	No. of patients	%	
Rhinitis	150	100	43	100.00	107	100.00	‐
Nasal obstruction	136	90.67	36	83.72	100	93.46	0.064
Rhinorrhea	130	86.67	38	88.37	92	85.98	0.697
Nasal pruritus	103	68.67	28	65.12	75	70.09	0.552
Sneezing	141	94.00	41	95.35	100	93.46	0.659
Conjunctivitis	139	92.67	41	95.35	98	91.59	0.424
Tearing	99	66.00	30	69.77	69	64.49	0.537
Ocular pruritus	126	84.00	38	88.37	88	82.24	0.354
Conjunctiva irritation	97	64.67	30	69.77	67	62.62	0.407
Asthma‐like symptoms	94	62.67	26	60.47	68	63.55	0.724
Cough	74	49.33	23	53.49	51	47.66	0.519
Chest constriction	44	29.33	10	23.26	34	31.78	0.300
Dyspnea	39	26.00	10	23.26	29	27.10	0.711
Wheezing	40	26.67	11	25.58	29	27.10	0.760
Skin reactions	39	26.00	8	18.60	31	28.97	0.191
Skin rash	27	18.00	5	11.63	22	20.56	0.198
Skin pruritus	31	20.67	7	16.28	24	22.43	0.400
Skin dryness	4	2.67	1	2.33	3	2.80	0.869

Clinical profiles	No. of patients	%
Rhinitis	2	1.3
Rhinitis + conjunctivitis	44	29.3
Rhinitis + asthma‐like symptoms	6	4.0
Rhinitis + skin reactions	1	0.6
Rhinitis + conjunctivitis + asthma‐like symptoms	59	39.3
Rhinitis + conjunctivitis + skin reactions	9	6.0
Rhinitis + asthma‐like symptoms + skin reactions	2	1.3
Rhinitis + conjunctivitis + asthma‐like symptoms + skin reactions	27	18.0

### 3.2 Ragweed pollen allergic patients show complex IgE reactivity profiles

Carbohydrate‐specific IgE levels of the 150 patients were measured using ImmunoCAPs containing ProGlycAn P. We found that 13.3% of the patients were positive, with ProGlycAn P‐specific IgE levels between 0.35 kUA/L and 24.00 kUA/L (Table S1).

Sera from the 150 ragweed allergic patients, serum from a non‐allergic person (NC) and buffer without serum (B) were analyzed for IgE reactivity to nitrocellulose‐blotted ragweed pollen extract, but only patients negative to CCDs by ImmunoCAP measurements (i.e., 130 patients) were considered for IgE sensitization profiles analysis, to reduce the role of CCD‐specific IgE. IgE reactivity profile of the CCD positive patients is available in Figure S1.

The IgE reactivity profiles of these 130 patients could be summarized into 19 different sensitization patterns P1‐P19 (Figure 1A). Seven distinct bands with different molecular weights were visible with different intensities and combinations. A 38–40 kDa double band occurred in all patterns except P19. At around 55 kDa, a band was visible in patterns P9, P12, P14, P15, P16, P17 and P18. A thick band around 24 kDa was observed in P3, P7, P10, P11, P12, P14, P16 and P17. Further, P2, P6‐P16 contained a band at around 15 kDa. Two other signals between 10 and 15 kDa appeared: a band near 10 kDa for P5, P8, P10, P12, P13, P14, P18, P19 and one at around 14 kDa for P4, P6, P11, P13, P14 and P15. Considering the number of bands, P14 is the most complex pattern displaying seven bands, followed by P12 with six and P10, P11, P13, P15 and P16 with five bands.

Interestingly, 13 patients displayed no IgE reactivity even after long exposure of the autoradiographies.

Serum from the non‐allergic individual (NC) and buffer without serum (B) showed no reactivity.

The protein composition of ragweed pollen extract was visualized by SDS‐PAGE and subsequent staining of proteins (Figure 1B). Bands can be observed at >55 kDa, approx. 40 kDa (two bands), 24 kDa, 14–15 kDa and a smear at 10 kDa. nAmb a 1.01 displayed three bands: one intense at approx. 40 kDa, a narrow band at 28 kDa and a faded band at 15 kDa. rAmb a 1.03 displayed only one intense band at around 38 kDa, lower compared to the one from nAmb a 1.01.

IgE reactivity to Amb a 1 was further tested using immunoblot inhibition experiments. Sera from 10 patients corresponding to different patterns: 7 (P6), 14 (P9), 35 (P8), 51 (P2), 54 (P13), 57 (P10), 64 (P9), 74 (P2), 81 (P7), 89 (P14) and displaying more than the two bands around 38–40 kDa were pre‐incubated with two Amb a 1 isoforms. Different levels and patterns of inhibition were observed (Figure 1C). nAmb a 1.01 inhibited IgE reactivity to the 38–40 kDa band (patients 14 and 54) and 15 kDa band (patients 7, 14, 51, 54, 64, 74, 81, 89), while for patients 7, 51, 57, 64, 74, 81, 89 only a partial inhibition could be observed. rAmb a 1.03 was able to totally inhibit the band at 15 kDa (patients 7, 14, 35, 51, 54, 64, 74, 81, 89) and only partially the bands at 38–40 kDa (patients 7, 14, 35, 51, 54, 57, 64, 74, 81, 89). IgE reactivity to 55 kDa, 24 kDa, 14 kDa and 10 kDa proteins could only be partially or not inhibited. For patient 89, a narrow band appeared at around 28 kDa and was inhibited by both nAmb a 1.01 and rAmb a 1.03.

Based on earlier work,^22^, ^42^, ^43^, ^44^ SDS‐PAGE with ragweed extract and Amb a 1 and IgE inhibition experiments, the double band from 38 to 40 kDa and the band at 15 kDa were considered as Amb a 1 (P1 and P2), but not the band at 24–28 kDa. This signal was not inhibited by Amb a 1 isoforms (Figure 1C) and may represent Amb a 4 (P3).^45^ According to these results, we have indicated in Figure 1A the possible nature of the different bands (Amb a 1, Amb a 3, Amb a 4, Amb a 5, Amb a 6, Amb a 7, Amb a 8, Amb a 9, Amb a 10, Amb a 11, Amb a 12) on the right side of the IgE immunoblot.

### 3.3 Association of IgE‐sensitization profiles with clinical symptoms

Patients with negative CCD‐specific IgE results were grouped according to IgE sensitization patterns and symptoms. The most frequent IgE‐sensitization patterns were P5 (16.9%) and P1 (11.5%) (Table S2). While pattern P1 includes bands belonging most likely to Amb a 1, pattern P5 includes bands that appear to belong to other ragweed allergens. Only three patients were not reactive to the double band at 38–40 kDa but to other ragweed allergens (P19). Ten percent of the patients displayed no visible IgE reactivity even after long exposure, indicating sensitization to IgE epitopes sensitive to denaturation (Table S2).

Among the patients with IgE reactivity on blot (i.e., 117 patients), 23.9% belonged to patterns P1 and P2, whereas the majority of patients (i.e., 76.0%) displayed reactivity to Amb a 1 and/or other ragweed allergens.

Stratification based on the number of reported symptoms showed that most of the patients had three (46.1%) or two (36.9%) symptoms (Figure S2).

**FIGURE 2:**
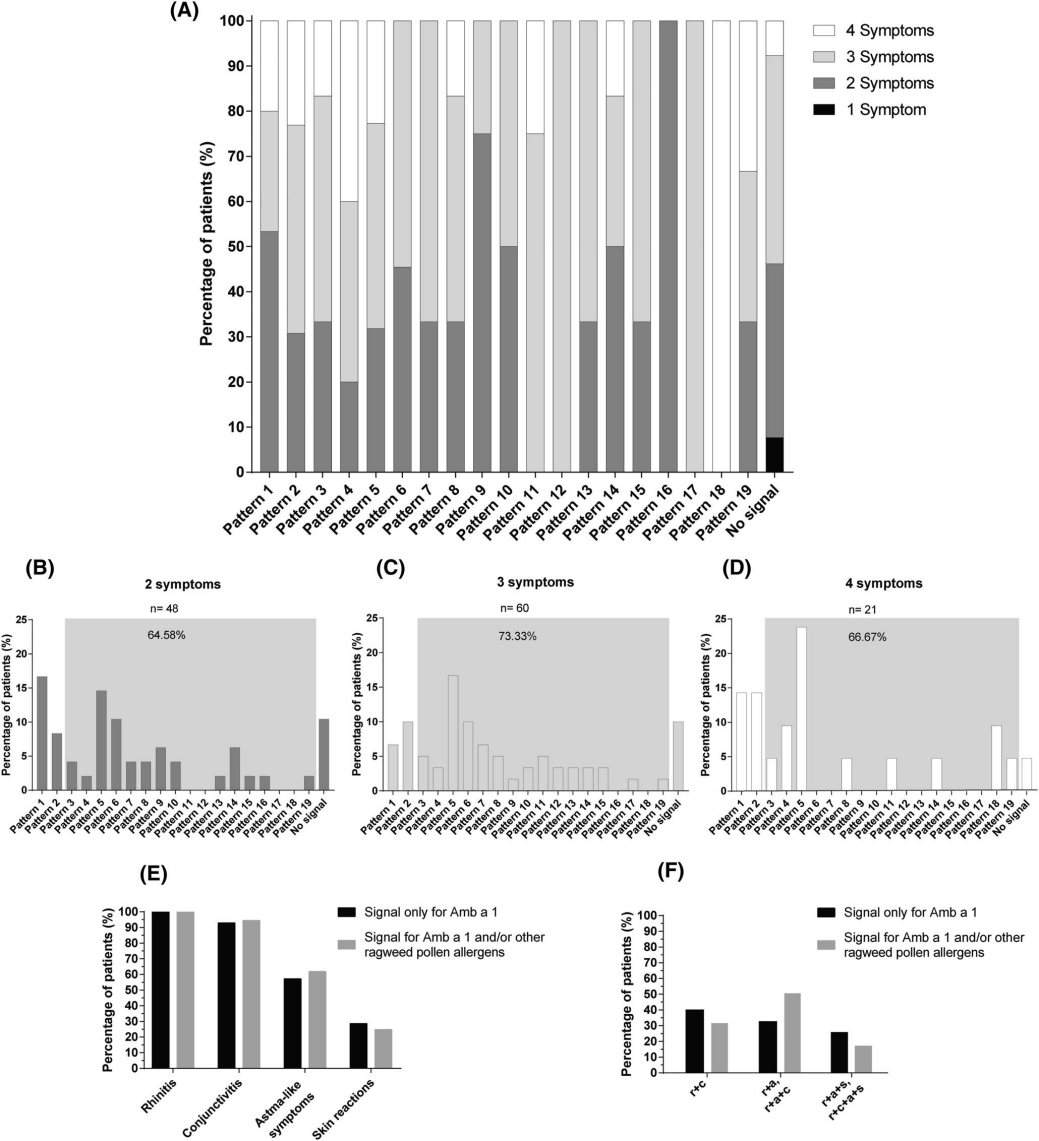
Association between IgE reactivity patterns and clinical symptoms. (A) Distributions of different numbers of reported symptoms (*y*‐axis: percentages of allergic patients) for each IgE reactivity pattern (*x*‐axis). Pattern distribution (*x*‐axis) of patients complaining of: (B) 2 symptoms, (C) 3 symptoms, (D) 4 symptoms (*y*‐axis: percentages of allergic patients) (Table S1). Gray boxes represent the percentage of patients with IgE reactivity not exclusively to Amb a 1. (E) Comparison of the symptom types (*x*‐axis) between patients with IgE reactivity only for Amb a 1 (black) and patients with IgE reactivity to Amb a 1 and/or other ragweed pollen allergens (gray) (*y*‐axis: percentages calculated from patients belonging to P1 and P2 (only Amb a 1, *n* = 28) and P3‐P19 (Amb a 1 and/or other ragweed pollen allergens, *n* = 89)). (F) Comparison of the symptom combinations (*x*‐axis) between patients with IgE reactivity only for Amb a 1 (black) and patients with IgE reactivity to Amb a 1 and/or other ragweed pollen allergens (gray) (*y*‐axis: percentages calculated from patients belonging to P1 and P2 (only Amb a 1, *n* = 27) and P3‐P19 (Amb a 1 and/or other ragweed pollen allergens, *n* = 81)). r + c‐rhinitis + conjunctivitis, r + a, r + a + c‐rhinitis + asthma and rhinitis + asthma + conjunctivitis, r + a + s, r + c + a + s‐rhinitis + astma + skin reactions, rhinitis + conjunctivitis + astma + skin reactions

Each IgE pattern was associated with a certain number of reported symptoms. Most of the patterns were associated with two or three symptoms (Figure 2A). For P5, one of the most frequent patterns, 31.8% of the patients reported two symptoms, 45.4% and 22.7% reported three and four symptoms, respectively.

When grouped according to the number of symptoms, 48 patients had two symptoms and 25% of these patients were sensitized mainly to Amb a 1 (pattern P1‐P2), while 64.5% were sensitized to Amb a 1 and/or to other ragweed allergens (patterns P3‐P19) (Figure 2B). This percentage increased to 73.3% for patients with three symptoms (*n* = 60) (Figure 2C) and was also high (i.e., 66.6%) for those with four symptoms (*n* = 21) (Figure 2D). Also, slightly more patients with Amb a 1 and/or other allergens sensitization reported conjunctivitis (94.3%) and asthma‐like symptoms (61.8%) when compared with patients sensitized only to Amb a 1 (92.8% conjunctivitis and 57.1% asthma‐like symptoms) (Figure 2E), but the difference was not statistically significant.

Among patients with different combinations of symptoms, a higher percentage showed reactivity towards Amb a 1 and other ragweed allergens. However, this difference in the proportion of patient reactivity within symptom groups was not statistically significant (Figure 2F).

### 3.4 Effects of Amb a 1‐specific IgE removal on basophil activation

Sera from patients 54 (pattern P13), 81 (pattern P7) and 89 (pattern P14) were subjected to depletion of Amb a 1.01‐ and 1.03‐specific IgE. They displayed signals on the blot corresponding to Amb a 1 and to other ragweed allergens (the band from 24 kDa and the two bands between 10 and 15 kDa) and showed total or partial inhibition with nAmb a 1.01 and rAmb a 1.03 (Figure 1C). Sera before and after IgE removal were tested by ELISA to demonstrate the reduction of specific IgE after allergen‐specific IgE depletion (Figure S3).

**FIGURE 3:**
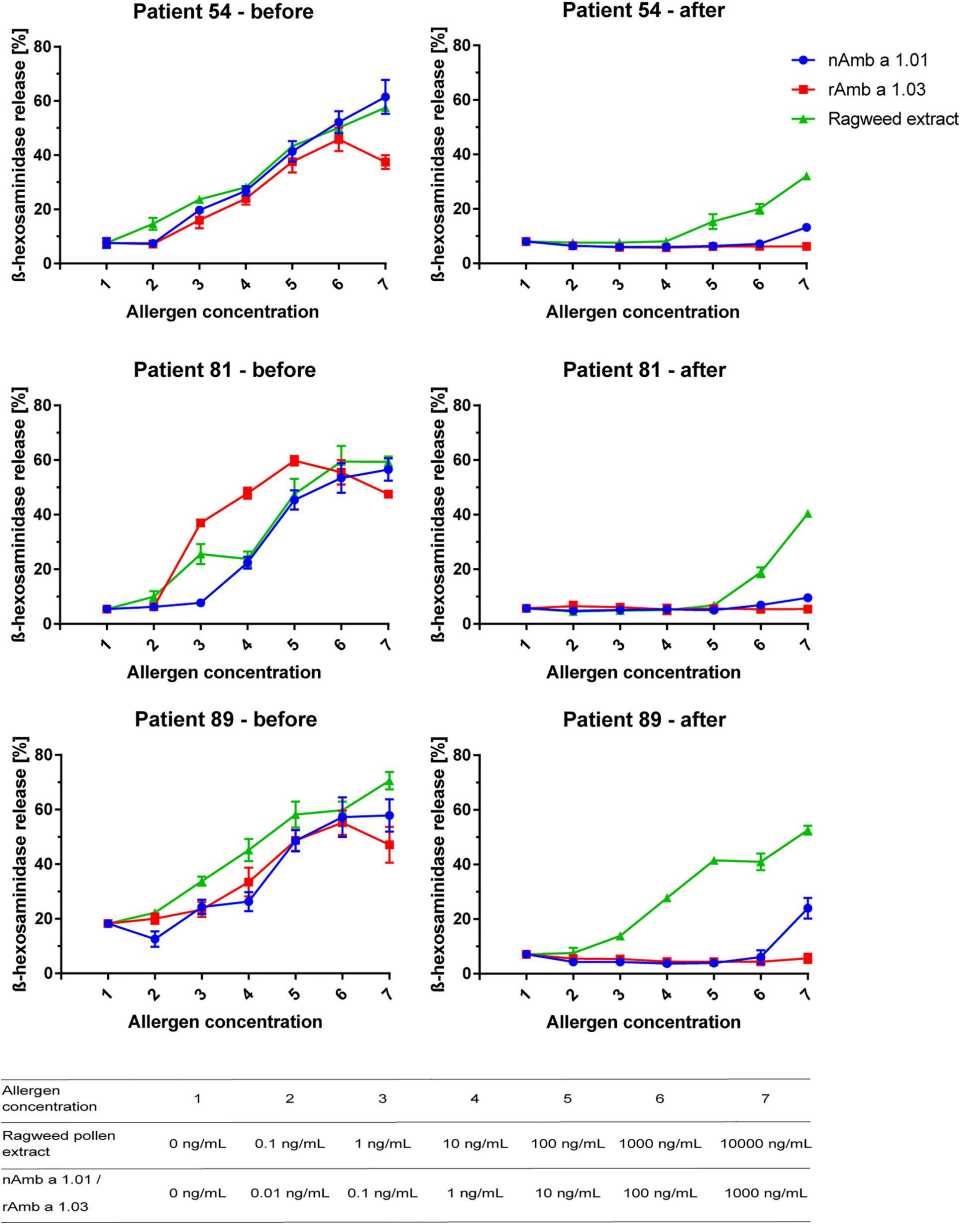
Comparison of the allergenic activity using sera before and after removal of Amb a 1‐specific IgE. Sera from three ragweed allergic patients, with confirmed IgE reactivity towards Amb a 1 and other ragweed pollen allergens before and after removal of Amb a 1.01‐ and Amb a 1.03‐specific IgE were loaded on rat basophil leukemia cells. Mediator release from RBL cells was triggered by serial dilution of nAmb a 1.01, rAmb a 1.03 and ragweed pollen extract (*x*‐axes). β‐hexosaminidase releases are expressed as percentages of total mediator contents +/− SD (*y*‐axes)

The allergenic activity of sera after Amb a 1‐specific IgE depletion was analyzed comparing sera before and after IgE removal in basophil activation experiments (Figure 3). Sera before IgE removal induced a strong concentration‐dependent mediator release when incubated with ragweed extract, nAmb a 1.01 or rAmb a 1.03 (Figure 3, left‐before). After IgE removal, sera were unable to induce degranulation for Amb a 1 isoforms but still induced degranulation with ragweed pollen extract (Figure 3, right‐after).

### 3.5 Identification of patients with strong IgE reactivity to CCDs in ragweed pollen

**FIGURE 4:**
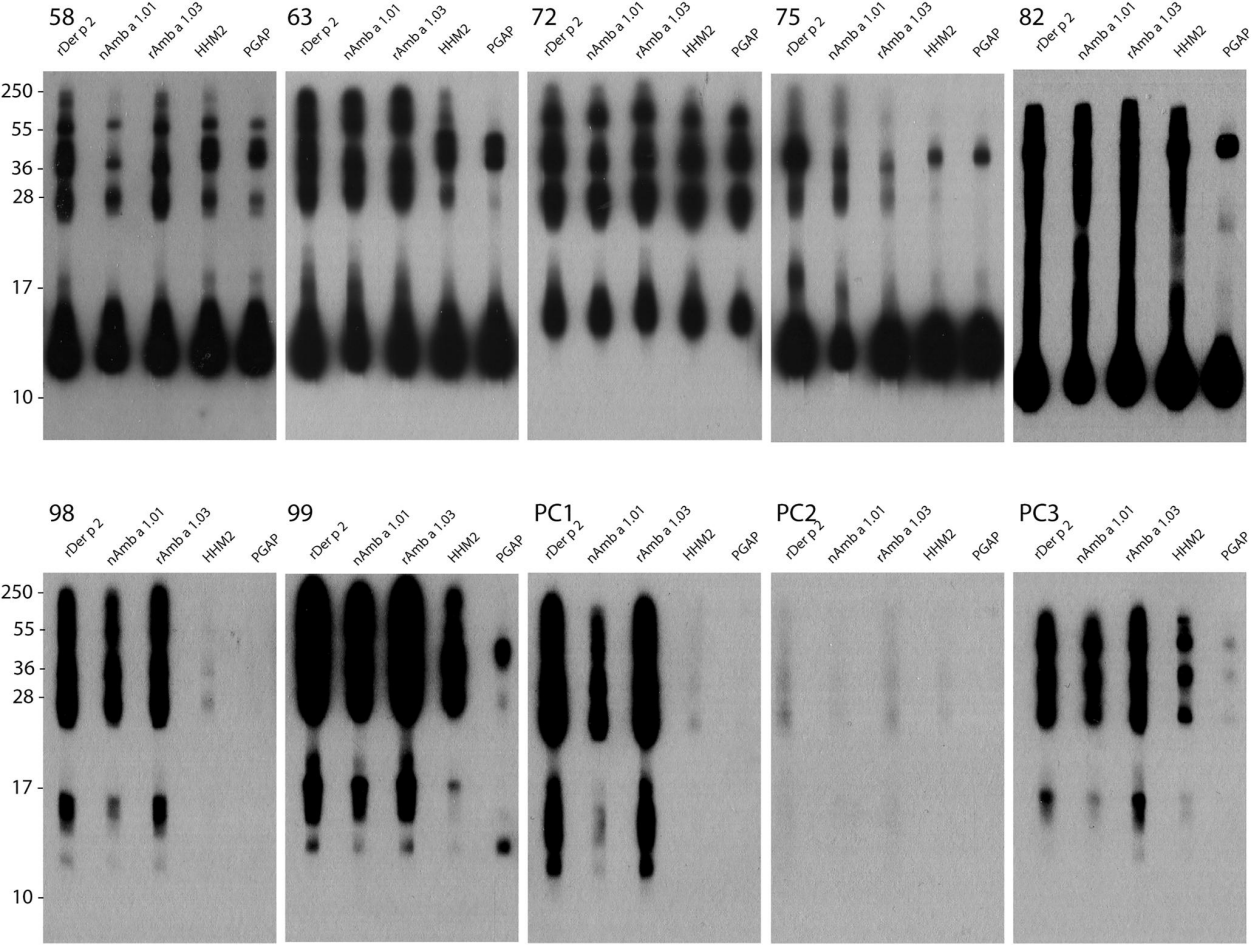
IgE immunoblot inhibition with nAmb a 1.01, rAmb a 1.03 and 2 CCD markers. Sera from seven ragweed allergic patients with CCD‐specific IgE and from three CCD‐positive subjects without ragweed allergy (PC1, PC2, PC3) were preadsorbed with rDer p 2, nAmb a 1.01, rAmb a 1.03 or two different CCD markers: recombinant horse heart myoglobin (HHM2) and ProGlycAn P (PGAP), and tested for IgE reactivity to nitrocellulose‐blotted ragweed pollen extract. Bound IgE antibodies were detected with ^125^I‐labeled anti‐IgE and visualized by autoradiography. Molecular weight (kDa) markers are indicated on the left

Sera from seven ragweed allergic patients positive to CCDs and three individuals sensitized to CCDs but not to ragweed (PC1‐3) were pre‐incubated with nAmb a 1.01, rAmb a 1.03 and two CCD markers and tested for IgE reactivity to nitrocellulose‐blotted ragweed pollen extracts. Pre‐incubation with HHM2 inhibited the ragweed extract‐specific IgE reactivity almost completely for patient 98, comparable with PC1 and PC2. Partial inhibition was observed for patients 63, 75, 82, 99 and PC3. ProGlycAn P inhibited IgE reactivity for patient 98, also for PC1 and PC2. Partial inhibition of IgE binding was observed for patients 58, 63, 75, 82, 99 and PC3 (Figure 4).

## 4 DISCUSSION

Ragweed pollen allergy represents a major health issue in the infested areas with increasing relevance due to climate change and urbanization.^13^, ^18^, ^19^ Currently, the determination of ragweed pollen‐specific IgE reactivity is based on extracts and, for molecular diagnosis, only Amb a 1 and Amb a 4 are available. Previously, Amb a 1 was considered the major ragweed allergen, sufficient for diagnosis and AIT of ragweed pollen allergy.^26^, ^27^ Meanwhile, 12 different ragweed pollen allergens and several Amb a 1 isoforms with distinct immunological features have been described.^2^, ^21^, ^22^, ^29^ Therefore, the goal of our study was to investigate the IgE reactivity profiles of a large representative and clinically well‐described population of ragweed pollen allergic patients, living in Western Romania, a region heavily affected by ragweed pollen allergy. The study was conducted as a pilot study using IgE immunoblotting and IgE inhibition to explore the contribution of purified Amb a 1 isoforms and CCDs to IgE epitopes in blotted allergen extracts. Furthermore, we analyzed possible associations between IgE reactivity profiles and clinical symptoms.

First, we analyzed symptoms related to ragweed pollen sensitization of 150 ragweed allergic patients (Table 1). Results showed that more than 95% of the patients suffered from rhino‐conjunctivitis, around 60% from asthma‐like symptoms and about 25% had skin reactions, in accordance with earlier studies reporting that ragweed pollen triggers rather respiratory symptoms than skin reactions.^46^, ^47^ Although not all the patients were monosensitized to ragweed, they reported severe symptoms only during the ragweed pollen season and no significant differences between mono‐ and polysensitized patients were observed (Table 1).

The analysis of IgE reactivity towards nitrocellulose‐blotted ragweed pollen extract in combination with IgE inhibition experiments allowed us to define 19 different IgE sensitization patterns in our cohort (Figure 1A). The most frequent signal was found at 38–40 kDa corresponding to Amb a 1 because it was inhibited by pre‐incubation of patients' sera with Amb a 1 (Figure 1C) similar to previous studies.^22^, ^43^, ^44^

It has been reported that Amb a 1 can undergo proteolysis forming two subunits (α‐ and β‐chain),^43^, ^48^ also observed by us (Figure 1B). The 38–40 kDa band represents the complete Amb a 1, the band around 28 kDa the α‐chain and the band at 15 kDa the β‐chain.

Interestingly, the two Amb a 1 isoforms showed different abilities to inhibit IgE binding to natural Amb a 1 in the blotted extracts as observed for patients 35, 54, 74 and 81 (Figure 1C) indicating different IgE epitopes in the two Amb a 1 isoforms. Partial inhibition of 38–40 kDa signal might suggest a sensitization to Amb a 11^30^ or to other Amb a 1 isoforms.

No inhibition could be observed for signals around 55, 24, 14 and 10 kDa, except a narrow band around 28 kDa (patient 89), representing probably the α‐chain of Amb a 1, indicating that these bands belong to other ragweed pollen allergens. The band around 55 kDa may be attributed to the newly discovered enolase Amb a 12 (48 kDa),^49^ the smear around 24 kDa may represent the defensin Amb a 4, although some studies place it at around 30 kDa,^31^ other studies observed a position on immunoblot similar with what we considered to be Amb a 4.^45^ The two broad bands below 15 kDa may comprise one or more ragweed pollen allergens such as 14 kDa profilin (Amb a 8),^50^, ^51^ two plastocyanins: at 11 kDa (Amb a 3)^52^ and 10 kDa (Amb a 7),^53^ a lipid transfer protein of 10 kDa (Amb a 6)^54^ or a polcalcin of 10 kDa (Amb a 9).^50^ Based on the reported IgE frequency of Amb a 8 and 6 and on the described amount of these allergens in the pollen,^42^, ^55^ we assume that the signal at around 14 kDa represents Amb a 8 and the one at 10 kDa represents Amb a 6.

The analysis did not reveal a dominant IgE recognition pattern. Pattern P5 including Amb a 1 and other ragweed pollen allergens (possibly Amb a 9 or Amb a 3, but most likely Amb a 6^42^, ^55^) was the most frequent IgE recognition pattern (16.9%) (Table S2). P1 and P2, considered as mainly Amb a 1‐reactive according to IgE inhibition experiments (Figure 1C), showed a frequency of 11.5% and 10.0% respectively. Interestingly, 10% of the patients showed no detectable IgE reactivity on blot suggesting that allergens with mainly conformational IgE epitopes, lost during denaturing SDS‐PAGE and blotting, may be involved, but a lack of signal due to low IgE level, therefore low sensitivity of the immunoblot test can not be excluded. Also patients without Amb a 1‐specific IgE were identified (Table S2). The percentage (12.3%) of patients with signal to other ragweed allergens than Amb a 1 (P19) (3 patients) and of those without signal on the blot (13 patients) is similar with the percentage (13.2%) of patients negative to Amb a 1 but positive to ragweed pollen extract skin prick test obtained by Haidar et al.^56^ in a population from the same geographic area.

Even patients belonging to P19 (Figure 1A), considered Amb a 1 negative, reported asthma‐like symptoms (Table S1), suggesting that other ragweed pollen allergens may induce these symptoms. Based on the existing data, at around 10 kDa can be found Amb a 9, Amb a 3, Amb a 7, but most likely the band represents Amb a 6, a better‐represented allergen in the ragweed pollen extract.^42^, ^55^

Analyzing the number of symptoms together with the IgE reactivity patterns it seemed that patients with complex sensitization profiles have more clinical symptoms (Figure 2A).

When considering all patients with two symptoms, 64.5% belonged to P3‐P19 with IgE reactivity to Amb a 1 and other ragweed allergens (Figure 2B). Of patients with three and four symptoms, 73.3% and 66.6%, respectively, belong to P3‐P19 (Figure 2C,D). A direct comparison regarding the type of symptoms showed that more patients sensitized to Amb a 1 and/or other allergens (P3‐P19) tended to have asthma‐like symptoms (Figure 2E,F), indicating that sensitization to other ragweed pollen allergens in addition to Amb a 1 may be associated with a higher number of symptoms and more often with asthma‐like symptoms. Similar results were observed for mugwort allergy where sensitization to three or more mugwort allergens resulted in a higher risk of allergic asthma.^57^ An increase in asthma risk was also observed in furry animal allergy studies for patients sensitized with more than one molecular component,^58^, ^59^ whereas, for grass pollen allergy, complex sensitization profiles were associated with longer disease duration.^60^, ^61^

The presence of clinically relevant allergens other than Amb a 1 relevant isoforms^22^ in ragweed pollen was indicated in basophil activation experiments performed with sera with and without Amb a 1.01 and 1.03‐specific IgE (Figure 3). According to the inhibition assays and literature data regarding the proportion of different allergens in ragweed pollen extract,^42^, ^55^ these clinically relevant allergens might be Amb a 4, Amb a 6, Amb a 8, Amb a 11.

CCDs can interfere in allergy diagnosis giving false‐positive results.^37^, ^62^, ^63^ Immunoblot inhibitions performed with CCD molecules demonstrated the presence of IgE‐reactive CCD in ragweed pollen extract and even identified subjects who were sensitized mainly to ragweed‐derived CCD epitopes.

In conclusion, our study identified in our population 19 different IgE reactivity patterns, showed different IgE reactivity to Amb a 1 isoforms and indicated that also other allergens in ragweed may be clinically relevant. It will therefore be important to perform a detailed analysis of IgE reactivity, basophil activation with ragweed allergen molecules and to analyze the association of molecular IgE reactivity profiles with symptoms. However, our study indicates that IgE reactivity profiles in ragweed pollen allergy are complex and several Amb a 1 isoforms and other ragweed pollen allergens are needed for molecular diagnosis and immunotherapy.

## AUTHOR CONTRIBUTIONS

**Maria‐Roxana Buzan**: Data curation; Lead, Formal analysis; Lead, Investigation; Lead, Methodology; Lead, Software; Lead, Visualization; Equal, Writing – original draft; Lead, **Lauriana‐Eunice Zbircea**: Formal analysis; Supporting, Investigation; Supporting, Methodology; Supporting, Writing – review & editing; Supporting, **Pia Gattinger**: Investigation; Supporting, Methodology; Supporting, Writing – review & editing; Supporting, **Elijahu Babaev**: Investigation; Supporting, Writing – review & editing; Equal, **Frank Stolz**: Investigation; Supporting, Writing – review & editing; Supporting, **Rudolf Valenta**: Conceptualization; Supporting, Methodology; Supporting, Resources; Supporting, Writing – review & editing; Supporting, **Virgil Păunescu**: Funding acquisition; Supporting, Resources; Supporting, Writing – review & editing; Supporting, **Carmen Panaitescu**: Investigation; Supporting, Project administration; Supporting, Resources; Supporting, Supervision; Supporting, Writing – review & editing; Supporting, **Kuan‐Wei Chen**: Conceptualization; Lead, Funding acquisition; Lead, Methodology; Equal, Project administration; Lead, Resources; Equal, Supervision; Lead, Writing – review & editing; Lead

## CONFLICT OF INTEREST

Rudolf Valenta has received research grants from HVD Life Biotech (Vienna, Austria) Viravaxx (Vienna, Austria), WORG Pharmaceuticals (Hangzhou, China) and serves as a consultant for Viravaxx and WORG Pharmaceuticals. Elijahu Babaev and Frank Stolz were working at the time of the study at Biomay AG, however, there are no direct conflicts with the data presented in this study. The remaining authors declare that the research was conducted in the absence of any commercial or financial relationships that could be considered a potential conflict of interest.

## Supporting information

Supporting Information S1

Supporting Information S2

Figure S1

Figure S2

Figure S3
