# Supplementary material for: Complex IgE sensitization patterns in ragweed allergic patients: Implications for diagnosis and specific immunotherapy
Source: Clin Transl Allergy. 2022 Jul 5;12(7):e12179. doi: 10.1002/clt2.12179 (PMC9254219; doi:10.1002/clt2.12179)
Supplement: Supplementary file 1 — Supporting Information S1 [file CLT2-12-e12179-s003.docx]

**Supplementary Material S1**

**Materials and methods**

*Patients’ sera*

Patients included in this study were recruited from February 2017 to May 2018 from an allergy centre in Timisoara, Romania, where they were clinically evaluated by an allergist. Further allergy tests were performed based on the anamnesis to confirm the suspected sensitization. From this patient pool, 150 ragweed-allergic patients were included in our study with a case history indicative of seasonal ragweed allergy, positive skin prick test or/and serum tests for ragweed-specific IgE. All recruited patients reported severe symptoms only during the ragweed pollen season although they were not all monosensitized to ragweed. Ragweed allergy-related symptoms were recorded based on anamnesis and a validated questionnaire following the criteria from ARIA (Allergic Rhinitis and its Impact on Asthma)^1^ and GINA (Global Initiative for Asthma)^2^. According to the reported symptoms, we defined the following clinical manifestations: rhinitis (nasal obstruction, rhinorrhea, nasal pruritus, sneezing), conjunctivitis (tearing, ocular pruritus, conjunctiva irritation), asthma-like symptoms (cough, chest constriction, dyspnea, wheezing) and skin reactions (skin rash, skin pruritus, skin dryness). The expression “asthma-like symptoms” was used to indicate that asthma-related symptoms (cough, chest constriction, dyspnea, wheezing) were recorded also from the patients that were not diagnosed with asthma at the moment of investigation.

The patients evaluation was performed by skin prick test (SPT) to various standardized cutaneous extracts of allergens (HAL, Düsseldorf, Germany): hazel (Corylus avellana), alder (Alnus incana), birch (Betula alba), plane (Platanus vulgaris), oak (Quercus robur), ash (*Fraxinus excelsior*), wheat (*Triticum aestivum*), rye (*Secale cereale*), barley (*Hordeum vulgare*), orchardgrass (*Dactylis glomerata*), lawn grass (Lolium perenne), timothy grass (Phleum pratense), grass pollen mix (orchard grass, lawn grass, red fescue, rye, timothy grass, meadow soft grass), mugwort (Artemisia vulgaris), ragweed (Ambrosia artemisiifolia), Alternaria alternata, Cladosporium herbarum, Aspergillus fumigatus, *Penicillium,* Candida albicans, dog (Canis familiaris), cat (Felis catus), house dust mites (Dermatophagoides pteronyssinus, Dermatophagoides farinae, tested separately), cockroach (Blatella germanica), egg, milk, walnuts, hazelnuts, peanuts, lemon, orange, banana, wheat flour, rye flour, barley flour, gluten, soy, celery, tomato, potato, chicken, pork, lamb, peas, beans, onion, paprika, peper, crustaceans, salmon, trout. Histamine dihydrochloride (10 mg/ml, equivalent to 6 mg histamine) was used as the positive control and a phenolated glycerol-saline solution as the negative control. The SPT was considered positive if the wheal diameter was at least 3 mm.

Allergen‑specific IgE antibodies in the blood were quantified by ImmunoCAP measurements (Thermo Scientific, Phadia AB, Uppsala, Sweden). Patients with IgE levels ≥ 0.35 kUA/L were considered positive.

Serum samples were collected from the patients after written informed consent was obtained. The usage of sera from ragweed allergic patients in this study was approved by the Local Ethics Commission of Scientific Research of the Pius Brinzeu Emergency County Hospital Timisoara (Ethical approval number 102, 10.01.2017). All experiments were performed following relevant guidelines and regulations.

*Pollen extract and allergens*

For aqueous extract preparation, 2 g of ragweed pollen (Allergon AB, Sweden) was shaken in 20 ml sterile DPBS (Gibco, Thermo Fisher Scientific, Inc., Waltham, MA, USA), pH 7.4, for 4 h at room temperature. Insoluble material was removed by centrifugation (20 000 xg, 30 min, 4°C). After centrifugation, the pollen extract was dialyzed against DPBS using Spectra/Por dialysis membrane with 3.5 kDa cut-off (Spectrum Labs, Repligen, CA, USA)^3^. Allergen extract was stored at -20° C until use.

Natural Amb a 1.01 (nAmb a 1.01) was purified from ragweed pollen extracts by standard chromatography, as described^4^.

Recombinant Amb a 1.03 (rAmb a 1.03) was produced by heterologous expression in *Pichia pastoris* and purified from culture supernatants, as described^4^.

Recombinant glycoprotein HHM 2 harbouring two N-glycosylation sites at the N-terminus of the non-allergenic monomeric protein horse heart myoglobin (HHM) was expressed in *Trichoplusia ni* (High Five) insect cells and isolated from culture supernatants using affinity chromatography, as described^5^.

Recombinant Der p 2 was expressed in *Escherichia coli* strain BL21 (DE3) and purified from inclusion bodies, under reduced conditions through affinity chromatography, as described^6^.

The total protein concentration of ragweed pollen extract and the concentrations of the allergens were determined by BCA assay (Pierce, Thermo Fisher Scientific, Inc., Waltham, MA, USA).

*ImmunoCAP measurement for CCDs*

Carbohydrate-specific IgE levels were quantified by ImmunoCAP measurements. ProGlycAn P (HÄMOSAN Life Science Services GmbH, Austria) was biotinylated and coupled to Streptavidin ImmunoCAPs (o121) (Thermo Scientific, Phadia AB, Uppsala, Sweden)^5^. ProGlycAn P was dialyzed against a carbonate buffer (0.1 M NaHCO_3_, 1 M NaCl) and incubated with a fivefold molar excess of biotin (Biotinamidohexanoyl-6-aminohexanoic acid N- hydroxysuccinimide ester, Sigma, St. Louis, MO, US) for 3 h. Excess of biotin was removed by dialyzing against DPBS. Prewashed Streptavidin ImmunoCAPs were loaded with 50 μL of the biotinylated ProGlycAn P (100 μg/mL) and incubated for 30 min at room temperature. IgE reactivity to ProGlycAn P was determined with Phadia®250 (Thermo Scientific, Phadia AB, Uppsala, Sweden. Patients with IgE levels ≥ 0.35 kUA/L were considered positive.

*Western-blot, SDS-PAGE and Immunoblot inhibition*

Ragweed pollen extract was separated on SDS-PAGE (18% SDS polyacrylamide gel), under reducing conditions using 500 µl of ragweed extract/gel.

Separated proteins were blotted onto 0.2 μm nitrocellulose membrane (Amersham Protran, GE Healthcare Life Science, Freiburg, Germany)^7^ and cut into 3 mm strips which were then blocked with buffer A (50 mM Na_2_HPO_4_, 0.6 mM NaH_2_PO_4_, pH 7.5, 0.5% v/v Tween-20, 0.5% w/v BSA, 0.05% w/v NaN_3_). Patients’ sera were diluted 1:10 with buffer A and incubated with the strips overnight (ON) at 4°C. After washing with buffer A, bound IgE was detected with ^125^I-conjugated anti-human IgE antibody (BSM Diagnostica, Vienna, Austria) and visualized by autoradiography^8^. Strips were exposed 10 days to Kodak X-OMAT LS films (Eastman Kodak Company, Rochester, NY, USA) using intensifying screens, at -70^°^C. Serum from a non-allergic individual and buffer without serum were used as negative controls.

Sera from all 150 patients were tested in immunoblot, but for the determination of the IgE sensitization patterns, only CCD negative patients were included (130 patients) (**Figure 1A**).

For the comparison between ragweed pollen extract and the Amb a 1 isoforms, four concentrations of extract (20 μg, 10 μg, 5 μg, 1 μg) and 1 μg of the two Amb a 1 isoforms (nAmb a 1.01, rAmb a 1.03) were separated under reduced conditions on 18% SDS-PAGE followed by Coomassie Brilliant Blue staining. The different concentrations of ragweed pollen extract were used for better visualization of different bands. The initial concentration (1 μg) was used for determining which bands appear even at low concentrations of extract.

For the inhibition experiment, patients' sera were diluted 1:10 in buffer A and pre-incubated overnight at 4°C with 5  μg/mL nAmb a 1.01 and rAmb a 1.03. For control purposes, patients’ sera were pre-incubated with a non-ragweed related allergen, rDer p 2 from house dust mite. Pre-adsorbed sera were incubated with nitrocellulose-blotted ragweed pollen allergen extract and bound IgE was detected as described above.

Ten ragweed allergic patients (7, 14, 35, 51, 54, 57, 69, 74, 81, 89) were selected for the inhibition experiment based on the different IgE profiles and number of bands they showed in the previous immunoblot experiment.

Another inhibition test was performed with seven CCD positive patients (58, 63, 72, 75, 82, 89, 99) from the study cohort determined by ImmunoCAP. These patients were selected based on the CCD specific IgE levels (IgE level greater than 3.5 kUA/L – Class 3 and above). The inhibition experiment was performed with two different CCD markers, the recombinant HHM2 and ProGlycAn P, as previously described. Sera from three non-ragweed allergic individuals but with carbohydrate-specific IgE were used as controls for CCD inhibition (PC1-PC3).

*Removal* *of Amb a 1.01 and 1.03-specific IgE*

Natural Amb a 1.01 and rAmb a 1.03 with a concentration of 5 µg/mL were incubated overnight at 4°C on 96-well plates (Maxisorp Nunc, Thermo Fisher Scientific, Inc., Waltham, MA, USA), washed twice with PBS + 0.05% Tween (PBST) and blocked with PBST + 3% BSA. Serum aliquots of 100 µL from three Amb a 1 allergic patients (54, 81, 89), negative to CCDs and co-sensitized to other ragweed allergens, were added first on nAmb a 1.01 coated plates and incubated successively for 3 h, 3 h, overnight, 2.5 h, 2.5 h, 2.5 h, 2.5 h, overnight, 3 h, 3 h, overnight and 3 h, at 4°C. The serum samples were then incubated in the same order on rAmb a 1.03 coated plates.

Depletion of Amb a 1.01 and Amb a 1.03-specific IgE was tested in ELISA. Natural Amb a 1.01 and rAmb a 1.03 (5 µg/mL) were coated overnight at 4°C on 96-well flat-bottom plates (Maxisorp Nunc, Thermo Fisher Scientific, Inc., Waltham, MA, USA). The plates were washed twice with PBS+0.05% Tween (PBST) and blocked for 2.5 h with PBST + 3% BSA, room temperature. The serum samples before and after antibody removal were diluted 1:5 in PBST + 0.5% BSA and incubated on the plates overnight at 4°C.

After five-time washing with PBST, bound IgE antibodies were detected with a 1:2500 diluted anti-human IgE horseradish peroxidase (HRP)-linked polyclonal antibody from goat (SeraCare, Milford, MA, USA) by incubating for 45 min at 37°C and 45 min at 4°C. After five times washing with PBST, colour development was performed by 100 µl/well addition of detection substrate 2,2′-Azino-bis(3-ethylbenzothiazoline-6-sulfonic acid) diammonium salt (ABTS) (Sigma Aldrich, St. Louis, MO, USA) in 60mM citric acid, 77 mM Na_2_HPO_4_ 2H_2_O and 3 mM H_2_O_2_. The absorbance was measured at 405 nm with reference at 490 nm on a microplate reader (Tecan Infinite M200 Pro, Grödig, Austria).

*Basophil activation assays*

To analyze the allergenic activity in Amb a 1 specific IgE depleted sera, rat basophil leukaemia (RBL) cell-release assays were performed. Rat basophil leukaemia cells (RS-ATL8)^9^ transfected with human FcεRI, kindly provided by Prof. Ryosuke Nakamura, were loaded with serum samples of ragweed allergic patients before and after Amb a 1.01- and Amb a 1.03-specific IgE removal. Serum samples were diluted 1:10 in MEM medium (Gibco, Thermo Fisher Scientific, Inc., Waltham, MA, USA) supplemented with 10% FBS (Gibco, Thermo Fisher Scientific, Inc., Waltham, MA, USA), penicillin-streptomycin 100 U/mL (Gibco, Thermo Fisher Scientific, Inc., Waltham, MA, USA), geneticin 0.2 mg/mL (Gibco, Thermo Fisher Scientific, Inc., Waltham, MA, USA), 0.2 mg/mL hygromycin B (Gibco, Thermo Fisher Scientific, Inc., Waltham, MA, USA), 0.2 mM L-Glutamine (Gibco, Thermo Fisher Scientific, Inc., Waltham, MA, USA) and incubated with the cells overnight at 37 °C. Cells were stimulated with serial dilutions of ragweed pollen extract (0.1 ng/ml - 10 µg/ml), nAmb a 1 or rAmb a 1.03 (0.01 ng/ml - 1 µg/ml). Buffer without allergens was used as a negative control. For 100% release, cells were lysed with 10%Triton-X. The release of β-hexosaminidase was measured with Varioskan LUX reader (Thermo Fisher Scientific, Inc., Waltham, MA, USA) and the results are shown as the percentage of total β-hexosaminidase release(100% release by addition of 10% Triton-X)^10^.

In order to have a comparable amount of allergens, a tenfold higher concentration of ragweed pollen extract, compared to Amb a 1, was used, based on the report that Amb a 1.01 and Amb a 1.03 represent 11.8% and 6.6%, respectively, from ragweed pollen extract^11^.

**References**

1. Brożek JL, Bousquet J, Agache I, et al. Allergic Rhinitis and its Impact on Asthma (ARIA) guidelines—2016 revision. *J Allergy Clin Immunol*. 2017;140(4):950-958.

2. Global Initiative for Asthma. Global Strategy for Asthma Management and Prevention, 2021. https://ginasthma.org/wp-content/uploads/2021/05/GINA-Main-Report-2021-V2-WMS.pdf. Accessed October 15 2021.

3. Egger C, Focke M, Bircher AJ, et al. The allergen profile of beech and oak pollen. *Clin Exp Allergy*. 2008;38(10):1688-1696.

4. Wolf M, Twaroch TE, Huber S, et al. Amb a 1 isoforms: Unequal siblings with distinct immunological features. *Allergy.* 2017;72(12):1874-1882.

5. Gattinger P, Mittermann I, Lupinek C, et al. Recombinant glycoproteins resembling carbohydrate-specific IgE epitopes from plants, venoms and mites. *EBioMedicine*. 2019;39:33-43.

6. Chen K-W, Fuchs G, Sonneck K, et al. Reduction of the in vivo allergenicity of Der p 2, the major house-dust mite allergen, by genetic engineering. *Mol Immunol*. 2008;45(9):2486-2498.

7. Towbin H, Staehelin T, Gordon J. Electrophoretic transfer of proteins from polyacrylamide gels to nitrocellulose sheets: procedure and some applications. *Proc Natl Acad Sci U S A*. 1979;76(9):4350-4354.

8. Valenta R, Duchene M, Ebner C, et al. Profilins constitute a novel family of functional plant pan-allergens. *J Exp Med*. 1992;175(2):377-385.

9. Nakamura R, Uchida Y, Higuchi M, et al. A convenient and sensitive allergy test: IgE crosslinking‐induced luciferase expression in cultured mast cells. *Allergy*. 2010;65(10):1266-1273.

10. Gieras A, Focke-Tejkl M, Ball T, et al. Molecular determinants of allergen-induced effector cell degranulation. *J Allergy Clin Immunol*. 2007;119(2):384-390.

11. Würtzen PA, Hoof I, Christensen LH, et al. Diverse and highly cross‐reactive T‐cell responses in ragweed allergic patients independent of geographical region. *Allergy*. 2020;75(1):137-147.
