# Supplementary material for: Complex IgE sensitization patterns in ragweed allergic patients: Implications for diagnosis and specific immunotherapy
Source: Clin Transl Allergy. 2022 Jul 5;12(7):e12179. doi: 10.1002/clt2.12179 (PMC9254219; doi:10.1002/clt2.12179)
Supplement: Supplementary file 2 — Supporting Information S2 [file CLT2-12-e12179-s001.docx]

**Supplementary Tables**

**Supplementary Table S1.** Clinical profile of each patient from the study population, grouped according to the ragweed IgE sensitization pattern determined in immunoblot and presence of carbohydrate-specific IgE determined in ImmunoCAP

| Pattern no. | Patient | Age | Sex | ProGlycAn P IgE levels  [kUA/L] | Rhinitis | Conjunctivitis | Asthma-like symptoms | Skin symptoms | Other sensitization than ragweed pollen |
| --- | --- | --- | --- | --- | --- | --- | --- | --- | --- |
| 1 | 1 | 47 | F | 0.00 | + | + | - | - | Wheat, Rye, Grass pollen mix , Cat, *Penicillium*, *Cladosporium*, *Alternaria*, *Aspergillus* |
|  | 4 | 23 | M | 0.00 | + | - | + | - | Grass pollen mix, HDM |
|  | 49 | 38 | F | 0.00 | + | + | + | + | - |
|  | 66 | 27 | M | 0.00 | + | + | - | - | - |
|  | 90 | 40 | M | 0.00 | + | + | + | - | Lawn grass, Wheat, Rye, Grass pollen mix |
|  | 93 | 24 | M | 0.00 | + | + | - | - | - |
|  | 104 | 35 | M | 0.00 | + | + | - | - | - |
|  | 109 | 33 | M | 0.00 | + | + | + | + | - |
|  | 124 | 30 | F | 0.01 | + | + | + | - | Grass pollen mix, HDM, Dog |
|  | 130 | 20 | F | 0.08 | + | + | + | - | HDM, Dog |
|  | 137 | 40 | F | 0.02 | + | + | + | - | HDM |
|  | 138 | 25 | F | 0.02 | + | + | - | - | - |
|  | 140 | 43 | F | 0.01 | + | + | + | + | - |
|  | 141 | 46 | F | 0.01 | + | + | - | - | HDM |
|  | 146 | 39 | M | 0.00 | + | + | - | - | Grass pollen mix, HDM, Cat |
| 2 | 11 | 29 | M | 0.02 | + | + | - | + | Mugwort, HDM, Cat |
|  | 22 | 39 | M | 0.00 | + | + | - | - | - |
|  | 24 | 32 | F | 0.00 | + | + | + | + | HDM |
|  | 26 | 40 | F | 0.00 | + | + | + | - | HDM, Mugwort |
|  | 51 | 28 | M | 0.10 | + | + | + | - | Lawn grass, Wheat, Rye, Grass pollen mix, HDM, Dog, Cat, *Candida* |
|  | 53 | 33 | F | 0.02 | + | + | + | + | Grass pollen mix |
|  | 61 | 29 | M | 0.00 | + | + | + | - | - |
|  | 68 | 32 | F | 0.00 | + | + | + | - | - |
|  | 74 | 35 | M | 0.02 | + | + | - | - | *Cladosporium* |
|  | 79 | 30 | F | 0.05 | + | - | + | + | Grass pollen mix, *Candida* |
|  | 84 | 45 | M | 0.04 | + | + | - | - | - |
|  | 88 | 30 | F | 0.01 | + | + | - | - | - |
|  | 111 | 31 | M | 0.00 | + | + | + | + | Grass pollen mix, Dog |
| 3 | 21 | 61 | M | 0.05 | + | + | + | - | Grass pollen mix, Mugwort, Dog |
|  | 34 | 29 | F | 0.00 | + | + | - | - | - |
|  | 45 | 49 | M | 0.00 | + | + | - | + | Mugwort, HDM |
|  | 47 | 37 | M | 0.00 | + | + | - | - | Hazel, HDM |
|  | 48 | 35 | M | 0.00 | + | + | + | - | Birch |
|  | 56 | 33 | F | 0.03 | + | + | + | + | Grass pollen mix, HDM |
| 4 | 10 | 45 | F | 0.01 | + | + | + | + | Wheat, Mugwort, HDM, Dog, *Candida* |
|  | 32 | 40 | M | 0.00 | + | + | - | - | - |
|  | 37 | 36 | M | 0.00 | + | + | + | - | Hazel, Birch, Ash, Lawn Grass, Rye, Wheat, Grass pollen mix, HDM, Dog |
|  | 132 | 48 | F | 0.01 | + | + | + | + | - |
|  | 147 | 25 | M | 0.02 | + | + | + | - | Hazel, Birch, Ash, Wheat, Grass pollen mix, HDM |
| 5 | 5 | 38 | M | 0.05 | + | + | - | - | Hazel, Lawn grass, Wheat, Rye, Grass pollen mix, Mugwort, Cat |
|  | 9 | 35 | M | 0.00 | + | + | + | - | HDM, Dog |
|  | 15 | 39 | M | 0.00 | + | + | - | - | HDM |
|  | 19 | 23 | M | 0.00 | + | + | + | - | HDM |
|  | 20 | 38 | M | 0.04 | + | + | - | - | - |
|  | 42 | 32 | M | 0.05 | + | + | + | + | Crustaceans |
|  | 67 | 18 | M | 0.08 | + | + | - | - | Wheat, Grass pollen mix |
|  | 69 | 33 | M | 0.00 | + | + | + | - | - |
|  | 73 | 43 | F | 0.00 | + | + | + | + | Grass pollen mix, Mugwort, HDM |
|  | 85 | 31 | M | 0.00 | + | + | + | + | - |
|  | 110 | 41 | F | 0.00 | + | + | - | - | - |
|  | 120 | 54 | M | 0.04 | + | + | + | + | Mugwort, HDM, Cat, *Alternaria* |
|  | 126 | 21 | M | 0.02 | + | + | + | - | HDM, *Candida*, *Blattella* |
|  | 127 | 38 | F | 0.01 | + | + | + | - | - |
|  | 128 | 20 | F | 0.01 | + | + | + | + | Egg, Peanut |
|  | 129 | 61 | F | 0.27 | + | + | + | - | Mugwort |
|  | 134 | 28 | M | 0.00 | + | + | + | - | HDM, Cat |
|  | 135 | 33 | M | 0.20 | + | - | - | + | Mugwort |
|  | 136 | 32 | F | 0.01 | + | + | + | - | Hazel, Mugwort, Dog |
|  | 142 | 37 | M | 0.22 | + | + | - | + | Hazel, Birch, Grass pollen mix |
|  | 143 | 28 | M | 0.01 | + | + | + | - | Wheat, Grass pollen mix |
|  | 150 | 48 | F | 0.02 | + | + | - | - | HDM |
| 6 | 2 | 31 | M | 0.00 | + | + | + | - | - |
|  | 3 | 30 | M | 0.02 | + | + | + | - | Ash |
|  | 7 | 33 | M | 0.11 | + | + | - | - | Ash, Hazel, Mugwort, Cat |
|  | 31 | 32 | M | 0.00 | + | + | - | - | Hazel, Birch, Ash, Rye, Wheat, Grass pollen mix, Mugwort |
|  | 50 | 45 | F | 0.01 | + | + | - | + | Birch, Wheat, Grass pollen mix, Mugwort, HDM, Dog, Cat |
|  | 52 | 36 | F | 0.00 | + | + | - | - | Hazel, Birch, Lawn grass, Wheat, Rye, *Cladosporium* |
|  | 76 | 35 | M | 0.17 | + | + | - | - | HDM |
|  | 80 | 49 | M | 0.10 | + | + | + | - | - |
|  | 94 | 35 | F | 0.00 | + | + | - | + | Lawn grass, Mugwort |
|  | 106 | 45 | F | 0.00 | + | + | + | - | Dog, Cat, *Aspergillus* |
|  | 125 | 31 | M | 0.00 | + | + | - | - | Mugwort |
| 7 | 8 | 37 | M | 0.00 | + | + | - | + | HDM, *Penicillium*, *Candida* |
|  | 12 | 26 | F | 0.00 | + | + | - | - | *Alternaria* |
|  | 23 | 32 | M | 0.01 | + | + | - | - | HDM, Mugwort |
|  | 55 | 29 | M | 0.00 | + | + | + | - | Ash, Lawn grass, Wheat, Rye, Grass pollen mix, Mugwort, HDM, *Alternaria* |
|  | 81 | 32 | M | 0.06 | + | + | + | - | - |
|  | 112 | 33 | M | 0.13 | + | + | + | - | Mugwort, HDM, Cat |
| 8 | 35 | 24 | F | 0.00 | + | + | - | - | *Alternaria* |
|  | 38 | 43 | M | 0.03 | + | + | + | + | HDM, Dog |
|  | 59 | 32 | M | 0.01 | + | + | + | - | HDM |
|  | 60 | 22 | M | 0.00 | + | + | + | - | - |
|  | 114 | 35 | M | 0.00 | + | + | + | - | - |
|  | 139 | 36 | M | 0.02 | + | - | + | - | Mugwort |
| 9 | 14 | 42 | M | 0.00 | + | + | + | - | Mugwort |
|  | 64 | 45 | M | 0.01 | + | - | + | - | Hazel, Wheat, Rye, Grass pollen mix, Mugwort, HDM, Dog, Cat, *Alternaria*, *Penicillium*, *Cladosporium* |
|  | 91 | 34 | M | 0.00 | + | + | - | - | - |
|  | 116 | 41 | F | 0.15 | + | + | - | - | *Aspergillus*, *Cladosporium* |
| 10 | 29 | 37 | M | 0.03 | + | + | - | - | *Alternaria* |
|  | 57 | 28 | M | 0.03 | + | + | + | - | - |
|  | 95 | 47 | M | 0.00 | + | + | - | - | - |
|  | 119 | 36 | M | 0.01 | + | + | + | - | - |
| 11 | 78 | 49 | F | 0.04 | + | + | + | + | Hazel, Birch, Egg |
|  | 102 | 59 | M | 0.00 | + | + | - | + | - |
|  | 113 | 49 | M | 0.01 | + | + | + | - | Wheat, HDM |
|  | 117 | 35 | M | 0.03 | + | + | + | - | Lawn grass, Grass pollen mix, HDM |
| 12 | 77 | 37 | M | 0.00 | + | + | + | - | Mugwort |
|  | 87 | 24 | M | 0.14 | + | + | + | - | - |
| 13 | 28 | 37 | F | 0.20 | + | + | - | - | Hazel, Birch, Rye, Cat |
|  | 54 | 43 | M | 0.01 | + | + | + | - | - |
|  | 115 | 34 | M | 0.03 | + | + | - | + | *Candida* |
| 14 | 62 | 40 | M | 0.00 | + | + | - | - | Lawn grass, Wheat, Rye, Grass pollen mix, Mugwort, HDM, *Aspergillus*, *Alternaria*, *Cladosporium* |
|  | 83 | 38 | M | 0.10 | + | + | + | + | - |
|  | 89 | 28 | M | 0.00 | + | + | + | - | Cat |
|  | 97 | 20 | M | 0.00 | + | + | + | - | Rye, Mugwort, HDM, Dog, Cat |
|  | 103 | 19 | F | 0.17 | + | - | + | - | HDM, Dog, Cat |
|  | 105 | 44 | F | 0.00 | + | + | - | - | Mugwort, HDM, *Alternaria*, *Candida* |
| 15 | 101 | 30 | M | 0.00 | + | + | + | - | Wheat, Dog, Cat, *Penicillium* |
|  | 108 | 29 | M | 0.10 | + | + | - | - | Mugwort |
|  | 122 | 33 | M | 0.03 | + | + | + | - | Mugwort |
| 16 | 86 | 32 | F | 0.02 | + | + | - | - | Wheat, Grass pollen mix, Mugwort, Cat |
| 17 | 30 | 45 | M | 0.00 | + | + | + | - | Hazel, Birch |
| 18 | 39 | 39 | M | 0.01 | + | + | + | + | Lawn grass, Wheat, Rye, Grass pollen mix, Mugwort, HDM, Dog, Cat, *Aspergillus*, *Alternaria*, *Penicillium*, Walnut, Hazelnut, Peanut, Banana, Orange, Apple, Barley flour, Wheat flour, Gluten, Salmon, Trout |
|  | 41 | 50 | F | 0.30 | + | + | + | + | Hazel, Lawn grass, Grass pollen mix, Mugwort, HDM |
| 19 | 17 | 36 | M | 0.00 | + | + | + | - | - |
|  | 46 | 31 | F | 0.00 | + | + | + | + | Hazel, HDM, Walnut, Peanut, Lemon, Potato |
|  | 133 | 42 | M | 0.00 | + | - | + | - | Hazel, Wheat, Rye, Grass pollen mix, *Aspergillus* |
| No signal | 13 | 33 | M | 0.04 | + | + | + | - | - |
|  | 16 | 37 | F | 0.01 | + | + | + | - | - |
|  | 18 | 39 | F | 0.00 | + | + | + | - | Hazel, Birch |
|  | 25 | 24 | F | 0.00 | + | + | - | - | - |
|  | 27 | 56 | M | 0.07 | + | + | + | - | Birch, Grass pollen mix, Rye, *Alternaria*, *Cladosporium* |
|  | 33 | 43 | F | 0.00 | + | + | + | - | *Alternaria*, Dog |
|  | 40 | 27 | M | 0.04 | + | - | - | - | Lawn grass, Wheat, Rye, Grass pollen mix, HDM, Cat |
|  | 43 | 25 | F | 0.00 | + | + | - | - | - |
|  | 96 | 24 | F | 0.00 | + | + | - | - | *Alternaria* |
|  | 131 | 27 | F | 0.02 | + | - | + | - | HDM |
|  | 144 | 26 | M | 0.24 | + | + | - | - | Wheat, Rye, Grass pollen mix, Mugwort, HDM |
|  | 145 | 44 | M | 0.00 | + | + | + | + | Wheat |
|  | 149 | 43 | M | 0.01 | + | + | + | - | Birch, Lawn grass, Grass pollen mix, Timothy grass, Barley, Orchardgrass, Mugwort, HDM, Dog, Cat |
| Positive to ProGlycAn P | 6 | 29 | M | 0.54 | + | + | + | - | Mugwort, HDM |
|  | 36 | 36 | M | 1.13 | + | + | + | + | Birch, Wheat, Rye, Grass pollen mix, Mugwort, HDM, Dog, Cat |
|  | 44 | 39 | M | 0.59 | + | + | + | - | HDM |
|  | 58 | 41 | M | 7.43 | + | + | - | + | Grass pollen mix, HDM |
|  | 63 | 35 | M | 19.60 | + | + | + | - | - |
|  | 65 | 36 | M | 0.35 | + | + | + | - | - |
|  | 70 | 34 | M | 0.91 | + | - | - | - | - |
|  | 71 | 33 | F | 1.31 | + | + | + | + | Birch, Lawn grass, Wheat, Rye, Grass pollen mix, Mugwort, Dog, *Penicillium* |
|  | 72 | 28 | M | 15.10 | + | + | + | - | Hazel |
|  | 75 | 37 | M | 7.92 | + | + | + | - | - |
|  | 82 | 48 | M | 4.07 | + | + | + | + | Hazel, Birch, Wheat, Grass pollen mix, *Candida* |
|  | 92 | 49 | M | 2.33 | + | + | - | - | Grass pollen mix |
|  | 98 | 47 | F | 5.35 | + | + | + | + | Birch, Grass pollen mix, HDM |
|  | 99 | 38 | F | 24.00 | + | + | - | - | Rye, Mugwort, HDM |
|  | 100 | 24 | M | 0.54 | + | + | + | - | Hazel, Wheat, Grass pollen mix, Mugwort, Dog, *Penicillium* |
|  | 107 | 61 | F | 0.40 | + | - | + | + | - |
|  | 118 | 44 | M | 0.48 | + | + | + | + | Wheat, Grass pollen mix, Mugwort |
|  | 121 | 39 | M | 0.72 | + | + | + | - | HDM |
|  | 123 | 35 | M | 1.27 | + | + | + | + | Mugwort |
|  | 148 | 37 | M | 0.35 | + | + | - | - | Grass pollen mix, Mugwort, HDM, Cat |

F – female; M – male; HDM - house dust mite; Grass pollen mix: Orchardgrass, Lawn grass, Red fescue, Rye, Timothy grass, Meadow soft grass

**Supplementary Table S2.** Demographic and clinical characterization of the 130 ragweed allergic patients with a negative response to CCDs in ImmunoCAP (patient distribution based on the IgE patterns determined in immunoblot)

| **Pattern** | **Patients displaying the pattern** | **Sex**  **(M/F)** | **Age** | **R** | **C** | **A** | **S** | **no. of**  **pat.**  **(%)** |
| --- | --- | --- | --- | --- | --- | --- | --- | --- |
|  |  |  | **Median [range]** |  |  |  |  |  |
|  |  |  |  | **no. of pat. (%)** | | | |  |
| **Pattern 1** | 1, 4, 49, 66, 90, 93, 104, 109, 124, 130, 137, 138, 140, 141, 146 | 7/8 | 35  [20-47] | 15 (100) | 14 (93.33) | 8 (53.33) | 3 (20.00) | 15 (11.54) |
| **Pattern 2** | 11, 22, 24, 51, 53, 61, 8, 74, 79, 84, 88, 111 | 7/6 | 32  [28-46] | 13 (100) | 12 (92.31) | 8 (61.54) | 5 (38.46) | 13 (10.00) |
| **Pattern 3** | 21, 34, 45, 47, 48, 56 | 4/2 | 36  [29-61] | 6 (100) | 6 (100) | 3 (50.00) | 2 (33.33) | 6 (4.62) |
| **Pattern 4** | 10, 32, 37, 132, 147 | 3/2 | 40  [25-48] | 5 (100) | 5 (100) | 4 (80.00) | 2 (40.00) | 5 (3.85) |
| **Pattern 5** | 5, 9, 15, 19, 20, 42, 67, 69, 73, 85, 110, 120, 126, 127, 128, 129, 134, 135, 136, 142, 143, 150 | 15/7 | 34  [18-61] | 22 (100) | 21 (95.45) | 14 (63.64) | 7 (31.82) | 22 (16.92) |
| **Pattern 6** | 2, 3, 7, 31, 50, 52, 76, 80, 94, 106, 125 | 7/4 | 36  [30-49] | 11 (100) | 11 (100) | 4 (36.36) | 2 (18.18) | 11 (8.46) |
| **Pattern 7** | 8, 12, 23, 55, 81, 112 | 5/1 | 32  [26-37] | 6 (100) | 6 (100) | 3 (50.00) | 1 (16.67) | 6 (4.62) |
| **Pattern 8** | 35, 38, 59, 60, 114, 139 | 5/1 | 33.50  [22-43] | 6 (100) | 5 (83.33) | 5 (83.33) | 1 (16.67) | 6 (4.62) |
| **Pattern 9** | 14, 64, 91, 116 | 3/1 | 41.50  [34-45] | 4 (100) | 3 (75) | 2 (50.00) | 0 (0.00) | 4 (3.08) |
| **Pattern 10** | 29, 57, 95, 119 | 4 0 | 36.50  [28-47] | 4 (100) | 4 (100) | 2 (50.00) | 0 (0.00) | 4 (3.08) |
| **Pattern 11** | 78, 102, 113, 117 | 3/1 | 49  [35-59] | 4 (100) | 4 (100) | 3 (75.00) | 2 (50.00) | 4 (3.08) |
| **Pattern 12** | 77, 87 | 2/0 | 30.50  [24-37] | 2 (100) | 2 (100) | 2 (100) | 0 (0.00) | 2 1.54) |
| **Pattern 13** | 28, 54, 115 | 2/1 | 37  [34-43] | 3 (100) | 3 (100) | 1 (33.33) | 1 (33.33) | 3 (2.31) |
| **Pattern 14** | 62, 83, 89, 97, 103, 105 | 4/2 | 33  [19-44] | 6 (100) | 5 (83.33) | 4 (66.67) | 1 (16.67) | 6 (4.62) |
| **Pattern 15** | 101, 108, 122 | 3/0 | 30  [29-33] | 3 (100) | 3 (100) | 2 (66.67) | 0 (0.00) | 3 (2.31) |
| **Pattern 16** | 86 | 0/1 | 32 | 1 (100) | 1 (100) | 0 (0.00) | 0 (0.00) | 1 (0.77) |
| **Pattern 17** | 30 | 1/0 | 45 | 1 (100) | 1 (100) | 1 (100) | 0 (0.00) | 1 (0.77) |
| **Pattern 18** | 39, 41 | 1/1 | 44.50 [39-50] | 2 (100) | 2 (100) | 2 (100) | 2 (100) | 2  (1.54) |
| **Pattern 19** | 17, 46, 133 | 2/1 | 36  [31-42] | 3 (100) | 2 (66.67) | 3 (100) | 1 (33.33) | 3 (2.31) |
| **No signal** | 13, 16, 18, 21, 25, 27, 33, 40, 43, 96, 131, 144, 145, 149 | 6/7 | 33  [24-56] | 13 (100) | 11 (84.62) | 8 (61.54) | 1 (7.69) | 13 (10.00) |
| **Total** | 130 patients | 84/46 | 35  [18-61] | 130  (100) | 121 (93.08) | 79  (60.77) | 31  (23.85) |  |

R – rhinitis symptoms, C – conjunctivitis symptoms, A – asthma-like symptoms, S – skin reactions, F-female,M - male

**Supplementary Figure legend:**

**Supplementary Figure S1** IgE reactivity patterns of ragweed allergic patients, positive to CCDs, to nitrocellulose-blotted ragweed pollen extract. Molecular weight (kDa) markers are indicated on the left.

**Supplementary Figure S2** Distribution of patients based on the number of symptoms. The frequency of ragweed pollen allergic patients (y-axis) reporting one, two, three and four symptoms (x-axis).

**Supplementary Figure S3** IgE reactivity after removal of Amb a 1-specific IgE towards Amb a 1 by ELISA. Shown are the IgE reactivities displayed in OD (y-axes) of sera from three ragweed allergic patients (patient 54, 81, 84) before and after Amb a 1.01- and Amb a 1.03-specific IgE removal (x-axes), buffer solution was used as a negative control.
