# Supplementary figures and images for: Complex IgE sensitization patterns in ragweed allergic patients: Implications for diagnosis and specific immunotherapy
Source: Clin Transl Allergy. 2022 Jul 5;12(7):e12179. doi: 10.1002/clt2.12179 (PMC9254219; doi:10.1002/clt2.12179)

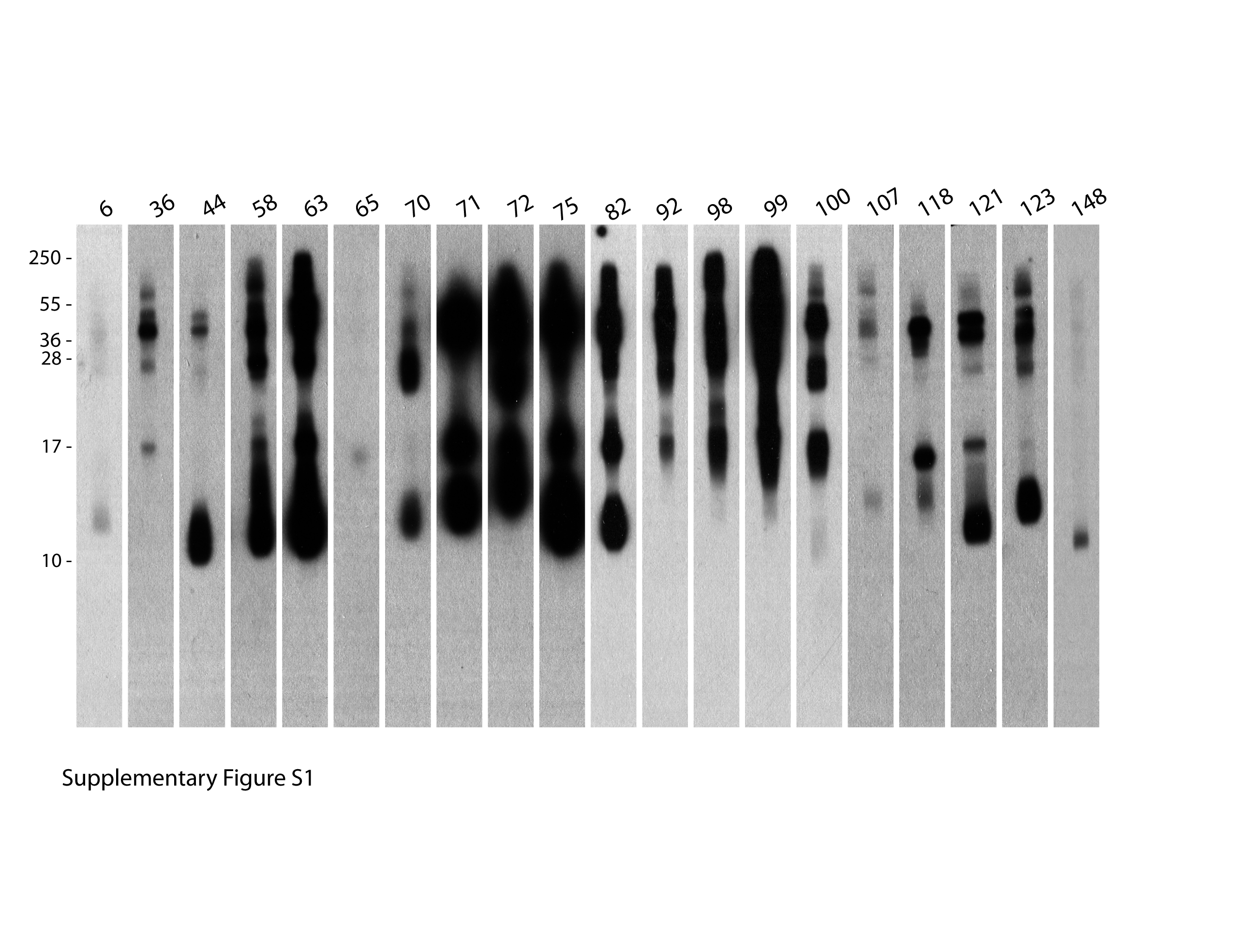

Supplement: Supplementary file 3 — Figure S1 [file CLT2-12-e12179-s002.tif]

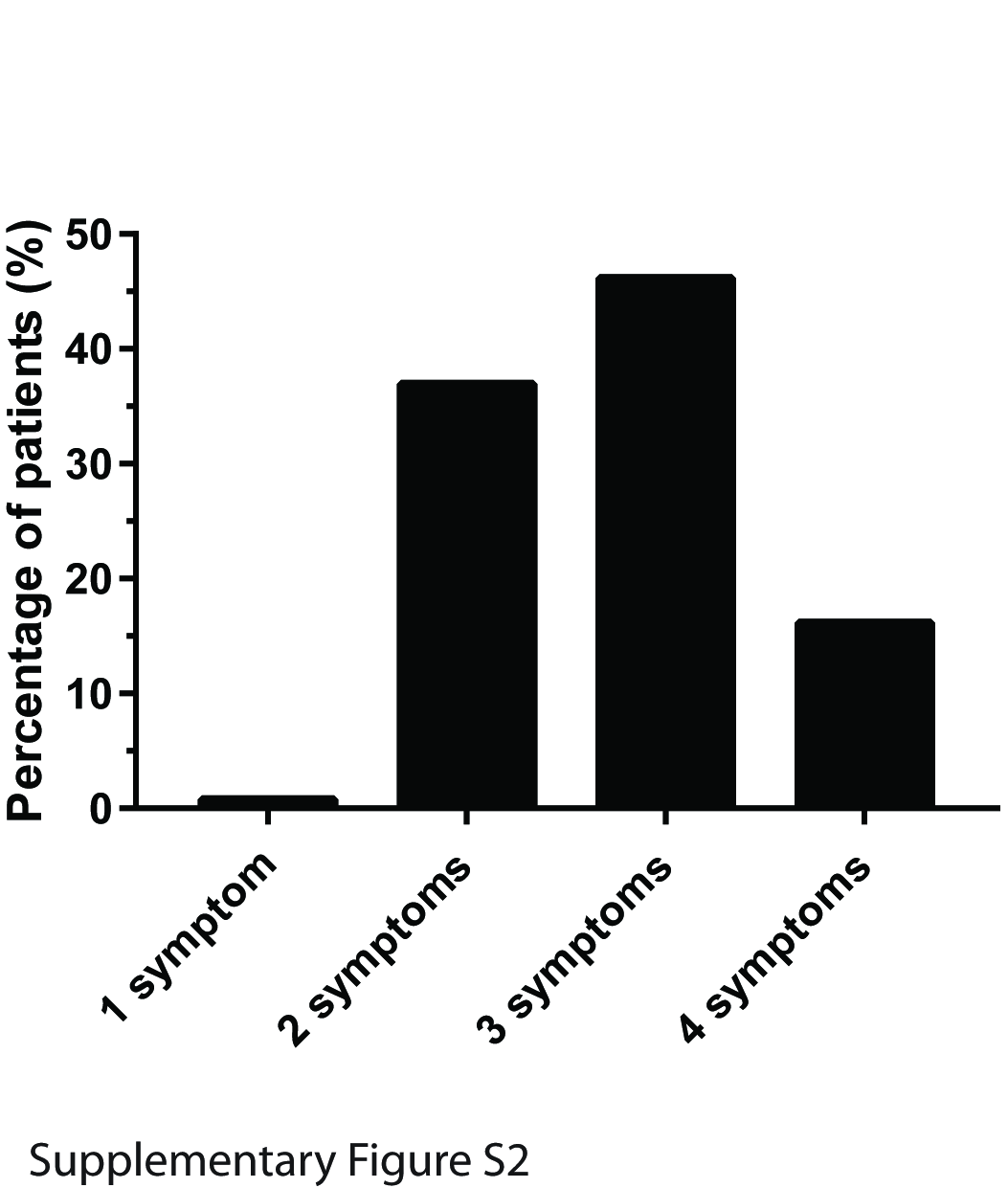

Supplement: Supplementary file 4 — Figure S2 [file CLT2-12-e12179-s004.tif]

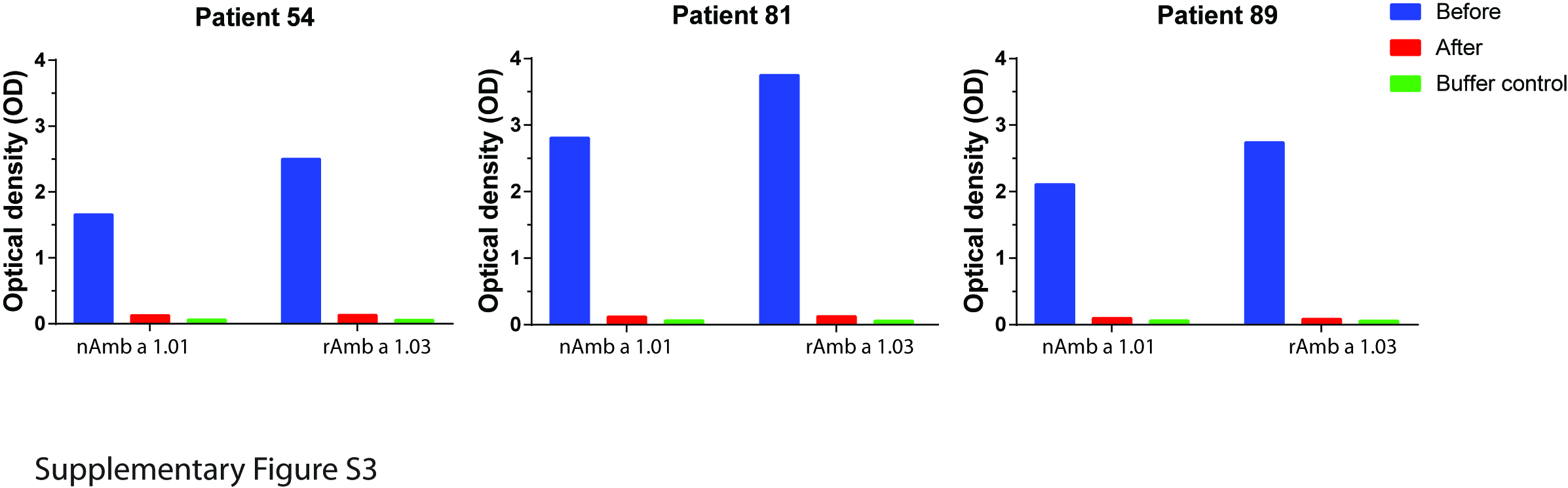

Supplement: Supplementary file 5 — Figure S3 [file CLT2-12-e12179-s005.tif]
